# Limitations to Accuracy in Extracting Characteristic Line Intensities From X-Ray Spectra

**DOI:** 10.6028/jres.107.045

**Published:** 2002-12-01

**Authors:** Peter J. Statham

**Affiliations:** Oxford Instruments Analytical Limited, High Wycombe, HP12 3SE, UK

**Keywords:** EDX, EDXS, energy dispersive, least squares fitting, microanalysis, spectrum processing, x rays

## Abstract

The early development of quantitative electron probe microanalysis, first using crystal spectrometers, then energy dispersive x-ray spectrometers (EDXS), demonstrated that elements could be detected at 0.001 mass fraction level and major concentrations measured within 2 % relative uncertainty. However, during this period of extensive investigation and evaluation, EDXS detectors were not able to detect x rays below 1 keV and all quantitative analysis was performed using a set of reference standards measured on the instrument. Now that EDXS systems are often used without standards and are increasingly being used to analyse elements using lines well below 1 keV, accuracy can be considerably worse than is documented in standard textbooks. Spectrum processing techniques found most applicable to EDXS have now been integrated into total system solutions and can give excellent results on selected samples. However, the same techniques fail in some applications because of a variety of instrumental effects. Prediction of peak shape, width and position for every characteristic line and measurement of background intensity is complicated by variations in response from system to system and with changing count rate. However, with an understanding of the fundamental sources of error, even a total system can be tested like a “black box” in areas where it is most likely to fail and thus establish the degree of confidence that should apply in the intended application. This approach is particularly important when the microanalysis technique is applied at lower electron beam voltages where the extraction of line intensities is complicated by extreme peak overlap and higher background levels.

## 1. Introduction

The technique of x-ray microanalysis relies on the fact that if a flat bulk sample and a standard are exposed to the same beam current and in the same geometry relative to an x-ray detector, then

$$C_{i}/C_{i}^{\text{std}}=XRCF*I_{i}/I_{i}^{\text{std}}$$ (1)

where *C*_i_ and *C*_i_^std^ are the mass concentrations of element i in sample and standard respectively, *I*_i_ and *I*_i_^std^ are the detected intensities of the characteristic line for element i in sample and standard and *XRCF* is the x-ray correction factor that accommodates the differences in x-ray generation, absorption, and fluorescence enhancement caused by the different overall compositions of sample and standard. In the two decades following Castaing’s 1951 thesis [1], x-ray intensities were invariably measured with high resolution Bragg crystal “wavelength dispersive” spectrometers (WDS) so it was straightforward to measure *I*_i_. In this period, *XRCF* calculations were extensively investigated and reported at international conferences. Consequently, relative uncertainties of 2 % could be expected for x-ray microanalysis of bulk materials in a dedicated WDS electron probe instrument [2].

When energy dispersive x-ray spectrometry (EDXS) systems were first introduced, the inferior instrumental resolution was a barrier to accurate measurement of line intensities and EDXS was only used as a rough qualitative tool. However, in 1973 Reed and Ware [3] demonstrated that quantitative analysis of silicate minerals for elements with atomic number 11 and above could be achieved by EDXS with limit of detection around 0.001 mass fraction and with an accuracy equivalent to WDS. While other authors subsequently corroborated these accuracy claims (e.g. [4]), a survey of EDXS accuracy on a variety of instruments [5] showed that relative standard uncertainties of 6 % could be expected for major constituents and much higher uncertainties could be obtained for concentrations below 0.2 mass fraction. This early report suggested that the source of error was primarily deconvolution of overlapping peaks and background correction.

Spectrum processing and EDXS instrumentation in general have undoubtedly improved since that survey, particularly in terms of convenience of use and operator deskilling. “Standardless” analysis has become popular because the requirement to maintain a collection of analytical standards for intensity comparison is a major overhead. Although some systems provide an option to use a single reference standard so that an analytical total can be obtained, most standardless procedures normalise the results. Although normalisation conveniently overcomes problems of beam current fluctuation, analytical errors are concealed beneath a total that is always unity. A recent report reviews the performance of some standardless EDXS solutions showing error histograms for analysis of a set of known materials [6]. While the commercial systems with fitted standards achieved relative standard deviations of around 12 %, a first principles approach gave a relative standard deviation of about 25 %. In the samples used, the *XRCF*’s were well known, all concentrations were above 0.05 mass fraction and most were above 0.20 mass fraction so the uncertainty due to background subtraction was not an issue. Standardless analysis requires more accurate spectrum processing and detector modelling because it does not benefit from the cancelling of first order error terms and implicit instrument calibration that occurs when ratioing measured intensities from sample and standard as in Eq. (1).

In the early work on quantitative EDXS and in the above assessment of standardless accuracy [6], the lowest energy line used was close to 1 keV. Most EDXS systems are now capable of detecting x rays well below 1 keV and the advantage of reduced analytical volume provides an increasing attraction to work at low beam voltages [7]. However, the poorer line energy separation, increased incidence of peak overlap, and higher background provide severe challenges for spectrum processing at these low energies. The low energy region also suffers electronic artifacts, incomplete charge collection (ICC) distortions of peak shape and large absorption edges in the background.

The successful early usage in mineralogical analysis, the sophisticated appearance of modern software, and the perceived security of a unity total provided by normalisation, have led to the expectation that all EDXS systems are capable of accurate quantitative analysis. However, with the increased application of standardless analysis, “out-of-the-box” with no customised installation, results are vulnerable to error, particularly when microanalysis is used outside the well-investigated territory of 1 keV to 10 keV energy and 15 kV to 25 kV accelerating voltage. This paper will demonstrate fundamental sources of error, how to avoid or overcome them and some approaches to validate overall spectrum processing performance for a “black box” system where the details of implementation are unknown to the operator.

## 2. Background Correction

### 2.1 Required Accuracy

**Fig. 1:**
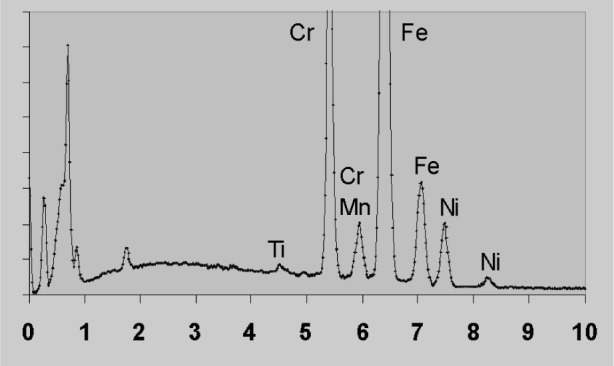
Spectrum of 465 steel sample recorded at 20 kV. Horizontal axis is energy in keV.

**Fig. 2:**
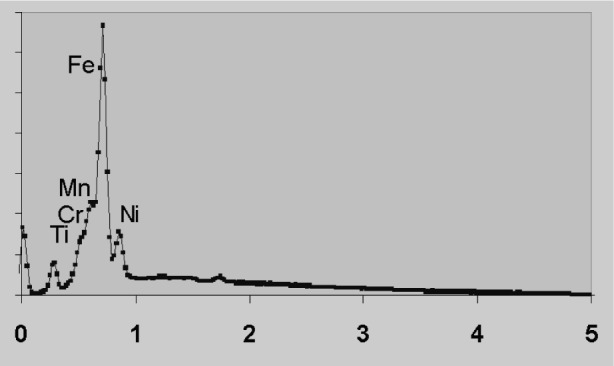
Same sample as for Fig. 1 recorded at beam voltage of 5 kV.

Spectrum processing requirements are illustrated by Fig. 1. At 20 kV, the K lines of Ti, Cr, Mn, Fe, Ni are excited and clearly visible in the spectrum between 4 eV and 9 keV. The background is fairly flat and there is some Kβ/Kα overlap to contend with, but most algorithms will have no difficulty in revealing Kα line intensities. However, Fig. 2 shows that when the same sample is excited at 5 kV, the spectrum processing task becomes formidable. The L line series for Ti, Cr, Mn, Fe, Ni all overlap, and the background bulges beneath the peaks so that a simple linear interpolation would give large percentage errors in composition.

**Fig. 3:**
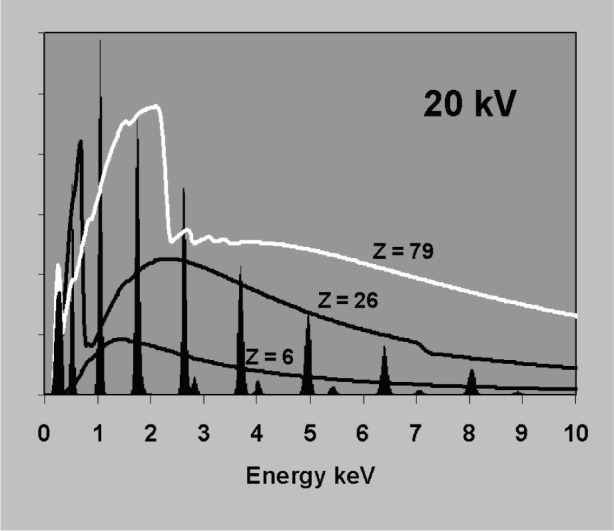
Bremsstrahlung background contribution detected at 40° takeoff-angle for a flat sample bombarded by 20 keV electrons. Backgrounds for pure C, Fe, and Au are shown (*Z* = 6,26,79) together with a series of characteristic K peaks for various elements showing the size of peak that is obtained at 0.01 mass fraction concentration for a Si(Li) detector with resolution 133 eV fwhm at 5.9 keV.

**Fig. 4:**
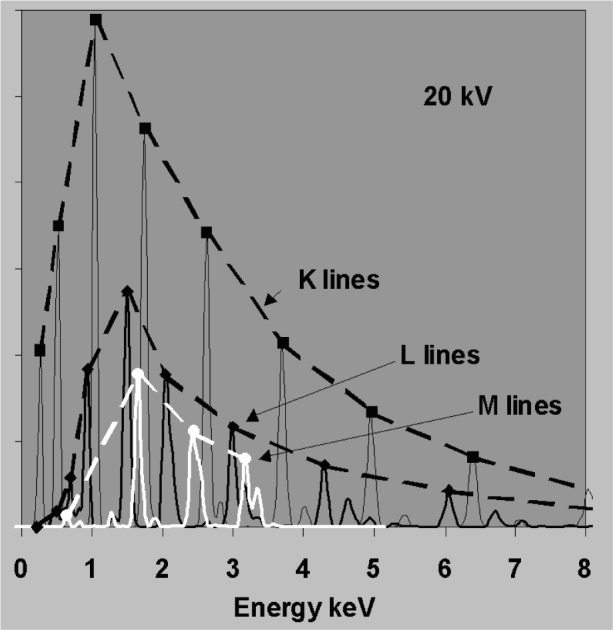
Relative intensities of x-ray lines excited in bulk pure elements at 20 kV for a Si(Li) detector with resolution 133 eV fwhm at 5.9 keV. The maximum peak intensity for K, L, and M series lines is shown as a function of x-ray energy to demonstrate that for the same concentration level, M and L lines are less intense than K lines.

**Fig. 5:**
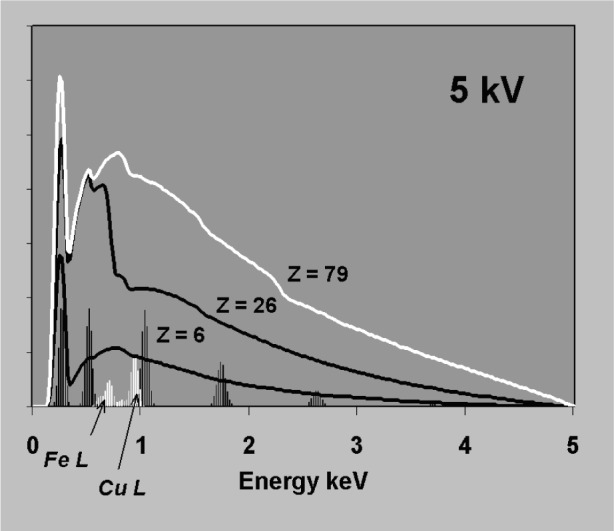
Bremsstrahlung backgrounds and characteristic peaks at 0.01 mass fraction concentration as for Fig. 3 but with a beam voltage of only 5 kV. Elements such as Fe and Cu can only be analysed using L lines at this voltage and the intensities equivalent to 0.01 mass fraction concentration are shown just below 1 keV.

The accuracy required in background correction can be understood with reference to Fig. 3. Whereas the bremsstrahlung background forms a smooth continuum with count rate per unit energy interval that is practically independent of detector resolution, the intensity within a single characteristic emission line is smeared into a peak with full width at half maximum (fwhm) determined by detector resolution so that the peak height is inversely proportional to resolution [see Eq. (7)]. The spectral response in Fig. 3 has been calculated for a typical Si(Li) detector with fwhm = 133 eV at 5.9 keV. Samples with higher atomic number generate proportionately more bremsstrahlung background and three examples are shown. Characteristic x-ray intensities depend mainly on element concentration and several K lines from different elements are shown at the 1 % level for reference. If the sample has a light matrix like C then the total background at any energy is much less than 1 % of the Kα peak height for a pure element. If the sample has a heavier matrix like Fe, then the background level represents about 0.01 mass fraction concentration and for samples with particularly heavy matrices like Au, the background can be equivalent to a mass fraction of several percent at higher energies. Therefore, to detect elements at the 0.001 mass fraction level using K lines, the estimate of background will usually have to be accurate to well within 10 %. If other lines are used, any error in the background correction has a greater effect because the peak height for L or M lines is less than for K at the same concentration, as shown in Fig. 4. At low kV, background correction has to be more accurate because the background represents a much higher mass fraction as can be seen by comparing Fig. 3 with Fig. 5. At 5 kV, for a sample with matrix atomic number of about 26, the background height at 2.6 keV is equivalent to about 0.03 mass fraction of ClKα and this is five times greater than at 20 kV. Moreover, fewer K lines are excited at low kV and lower intensity L lines have to be used. Now the background height from an Fe matrix is equivalent to almost 0.10 mass fraction concentration for FeL. At 5 kV beam voltage, a background correction accurate to within 1 % is therefore required to detect elements at the 0.001 mass fraction level using L lines. The bremsstrahlung shape for Fe in Fig. 5 also demonstrates the type of large step over the LIII absorption edge that makes background prediction particularly difficult at low energies.

### 2.2 Interpolated Background

**Fig. 6:**
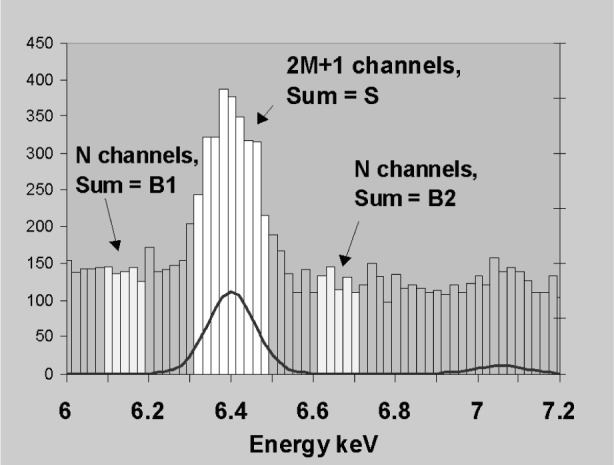
Digitised spectrum showing FeKα and FeKβ peaks superimposed on bremsstrahlung background. Channels used for computing background and peak intensity for FeKα are highlighted.

Interpolation and extrapolation techniques are described in detail in Ref. [8]. At higher energies where the background is fairly linear, subtracting an interpolated background is a reliable method to determine the area of an isolated peak. Figure 6 shows one approach that simplifies the equations. If we take the sum of (2*M* + 1) channels straddling the peak and define two background regions of *N* channels exactly the same distance away from the peak centre, then the formula for the net window integral, *P*, is:

P=S−(B1+B2)⋅(2M+1)/(2N). (2)

The standard deviation, *σ_P_* is given by

$${\sigma_{P}}^{2}=S+(B1+B2)\cdot {\left[(2M+1)/(2N)\right]}^{2}.$$ (3)

and this is minimised by choosing a large number of background channels *N*. In practice it is not easy to extend *N* indefinitely because of nearby peaks. In Fig. 6 the background window clearly needs to be far enough away from the nearby FeKβ peak just above 7 keV. The net integral *P* is proportional to peak area but the proportionality constant cancels when making comparisons from sample and standard as in Eq. (1). However, for standardless analysis, the proportionality constant has to be determined and will be altered if detector resolution changes for any reason.

### 2.3 Digital Filtering

Rather than measuring the net window integral, the average peak intensity over (2*M* + 1) channels can be used and the average background subtracted thus:

$$P^{\prime }=S/(2M+1)-(B1+B2)/(2N).$$ (4)

**Fig. 7:**
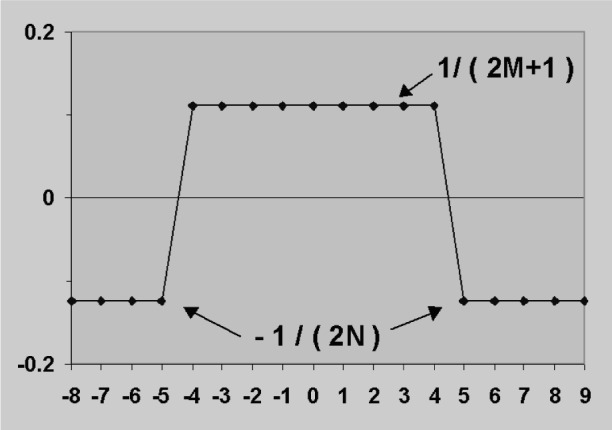
Weighting coefficients at each channel position for a “Top-Hat” digital convolution filter.

**Fig. 8:**
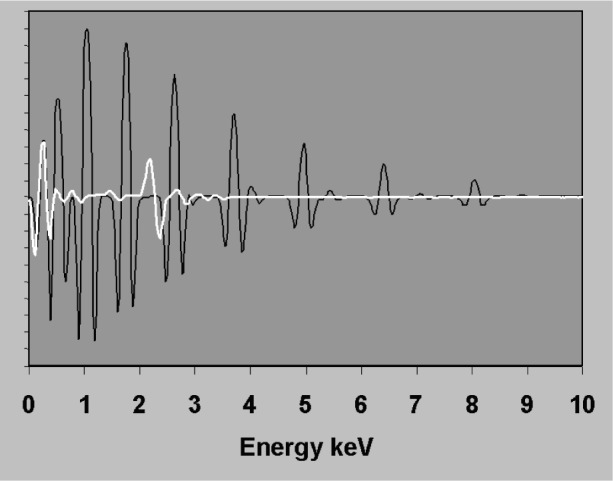
Result of digital filtering the data from Fig. 3 using the Top-Hat of Fig. 7. The K peaks at 0.01 mass fraction are converted into bipolar shapes. The white curve shows the filtered background for Au, *Z* = 79. No other backgrounds are shown.

This can be regarded as a weighted sum of channel counts where the channels over the peak are each multiplied by 1/(2*M* + 1) and the background channels multiplied by – 1/(2*N*) before summing. If the peak channels touch the background channels, then a graph showing the positive and negative weighting coefficients has a “Top-Hat” shape (Fig. 7). This “Top-Hat” can be positioned anywhere in the spectrum to calculate the net peak intensity at that position. If this is done for every channel position in the spectrum, the net peak intensity at each channel is effectively the output of a digital convolution filter. If 2*M* + 1 is chosen to be close to the fwhm of a peak and 2*N* covers roughly the same number of channels, then the filter will remove any background that is linear over the range of the top hat and will emphasise peaks. Figure 8 shows the result of running such a filter along the biggest of the background curves shown in Fig. 3. The K peaks at 0.01 mass fraction concentration have also been filtered the same way and it is clear that the residual background after filtering is equivalent to much less than 0.001 mass fraction everywhere in the spectrum except in the vicinity of the AuM absorption edge near 2 keV and the AuN edges below 0.5 keV. The filtering operation converts Gaussian peaks into peaks with negative side lobes but the filtered shapes can still be used in a least squares fitting procedure [9,10]. No background points have to be selected, the method is immune to any smooth background artifact such as backscattered electrons or pile up continuum and no prior knowledge of sample, geometry or microscope parameters are needed in order to make a background correction. The technique therefore has widespread applicability and has been used successfully in commercial EDXS systems for more than 25 years.

### 2.4 Background Modelling

A drawback with the digital filtering approach is that it cannot separate high background curvature from the curvature of a peak and this is particularly apparent in the vicinity of absorption steps as demonstrated in Fig. 8. Even in this rather extreme case, the measured peak will only suffer a maximum 0.003 mass fraction equivalent error if it is in a particular energy position relative to the AuM absorption edge. Nevertheless, accuracy can sometimes be improved by exploiting more prior knowledge of the background shape.

If the sample is flat, homogeneous, and semi-infinite, the beam voltage is known and an estimate of composition of the specimen is available, then the theoretical bremsstrahlung background, *B_E_*, for a single channel at energy *E*, can be calculated by including terms for sample and detector effects:

$$B_{E}={\left(\text{detector}\;\text{efficiency}\right)}_{E}\cdot {\left(\text{absorption}\right)}_{E}\cdot {\left(\text{generation}\right)}_{E}.$$ (5)

**Fig. 9:**
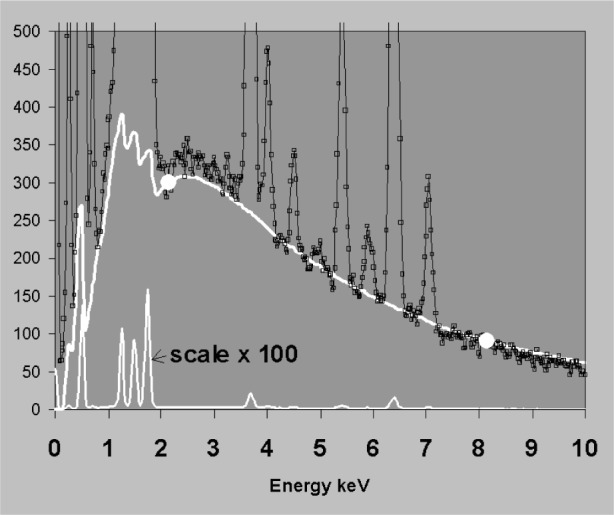
Spectrum of pyrope garnet obtained at 20 kV. White curve shows theoretical background modified to fit the spectrum at 2.16 keV and 8.14 keV. Absorption steps for Mg, Al, and Si are visible in the background between 1 keV and 2 keV.

**Fig. 10:**
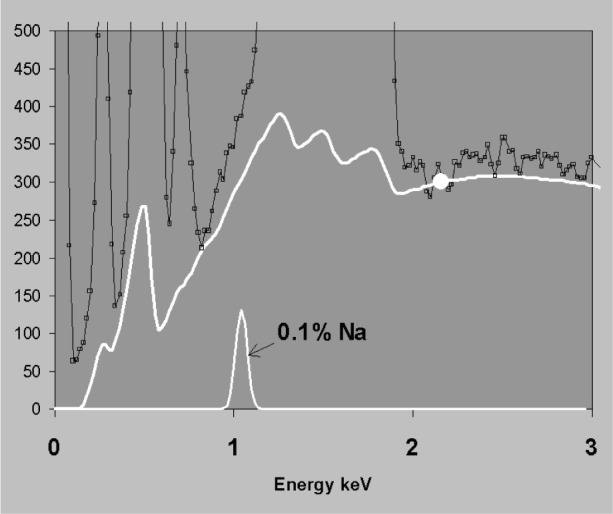
Enlarged view of Fig. 9 shows that there are no peak-free points for background fitting below 2 keV. The extrapolated background suggests the presence of Na with a mass fraction of 0.01 although this is not expected in the standard sample used.

After computing *B_E_* for every channel in the spectrum, the result is convolved with a Gaussian function where fwhm varies according to the detector resolution as a function of energy (see Sec. 3.3). Detector efficiency is straightforward to calculate but is a source of error because usually only nominal data are available for window thickness and composition, there are manufacturing variations from window to window and there may be additional absorption if a thin layer of oil has condensed on the window, or ice has built up on the crystal surface. The other major source of error is in the generation term. Various authors have shown that the standard “Kramers” generation term *K*_1_ · *Z* · (*E*_0_ −*E*)/*E*, where *K*_1_ is a constant, *Z* is the mean atomic number of the specimen, *E*_0_ is the energy of incident electrons, and *E* is the energy of the radiation, does not fit observed spectra very well. Lifshin [11] found that adding an additional quadratic term. *K*_2_ · *Z* · (*E*_0_ − *E*)^2^/*E* provided a considerably better fit to the distribution as a function of energy. This observation was exploited in the popular “FRAME” public domain software where measurements at two background energies were used to determine *K*_1_ and *K*_2_ [12] and this has also been adopted in some commercial EDXS systems. If the “Kramers” term is regarded as the “theory” then including a quadratic term is equivalent to modifying the theory with a multiplier that varies with energy thus:

$$\text{Modelled background}=B_{E}\cdot (a+b\cdot E)$$ (6)

where *a* and *b* are constants which force the theoretical *B_E_* to match the observed background at two chosen energies. This procedure can then be used to improve the fit for any theoretical bremsstrahlung formulation. Figure 9 shows a theoretical background that has been fitted to a spectrum of pyrope garnet at 2.16 keV and 8.14 keV. The fit between 3 keV and 8 keV is clearly very good and in general the modelling approach works well above 3 keV where absorption effects are slight. At lower energies uncertainty in detector efficiency is a problem and the correct estimation of absorption effects, (absorption)*_E_* in Eq. (5), requires the composition to be known and this has to be determinated by iteration. To do this, major elements must first be correctly identified and if a peak from an element like Si is misidentified as a much heavier element like W or Ta, then the calculated background shape will be incorrect. Another difficulty is that peak-free background points have to be found for fitting, ideally either side of every cluster of peaks. To identify whether a peak is present typically requires a region at least 4 × fwhm in width to do a background subtraction as in Fig. 6. At low energy, such regions are rarely available so the background has to be extrapolated from a fit at higher energies. In the straightforward example shown in Fig. 10, the background at Na is underestimated. Fitting at 2.5 keV would raise the level, and the error in predicting the background would exceed the statistical uncertainty at Na.

**Fig. 11:**
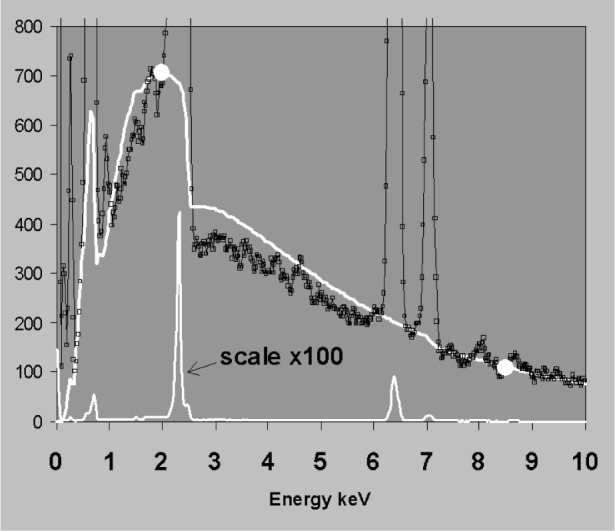
Spectrum from sample of pyrite, FeS_2_, obtained at 20 kV. Background model is fitted to background regions near 2 keV and 8 keV and shown by the thick white line.

**Fig. 12:**
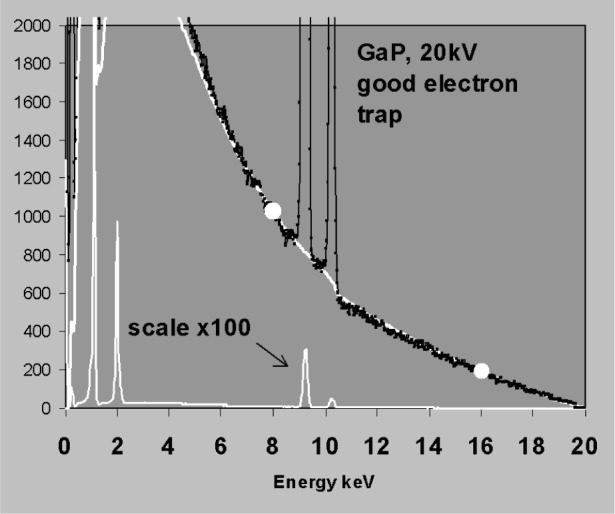
Spectrum from Gallium Phosphide standard obtained at 20 kV. White curve shows background model fitted to points near 8 keV and 16 keV.

**Fig. 13:**
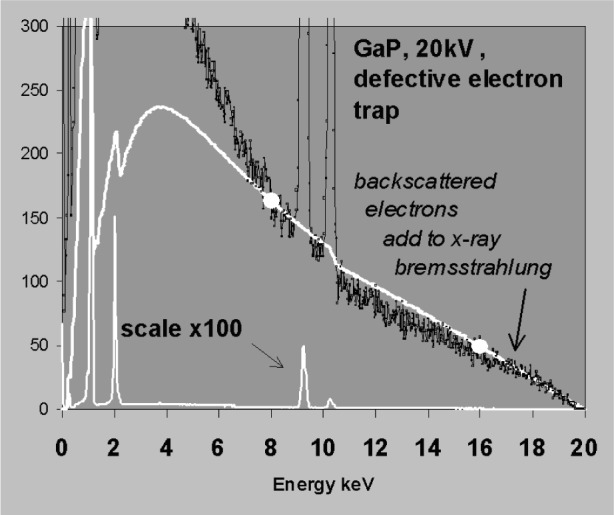
Same as for Fig. 12 but the spectrum is recorded on a detector with a defective electron trap.

The points chosen for background fitting must be representative of the underlying bremsstrahlung background. Figure 11 demonstrates a bad fit to a spectrum of FeS_2_ where the background has been fitted using points at 2 keV and 8 keV. The point at 2 keV is raised above the true background by a residual low energy tail on the SKα peak caused by incomplete charge collection (ICC); consequently the fit is poor both at low energies and above the SK peak. Whereas tailing is not present on every peak, this example demonstrates that difficulties arise with background modelling whenever there are smooth background artifacts that are not detected as peaks. In Fig. 12, the background model for the GaP spectrum fits well at all energies when points at 8 keV and 16 keV are used for scaling. In contrast, Fig. 13 shows a spectrum recorded from the same sample under the same conditions but using a detector with a defective electron trap. This time, when the theoretical model is force fit to the spectrum using the same points at 8 keV and 16 keV, the background estimate is very poor. In this case, the continuum due to backscattered electrons that enter the detector has raised the background at 16 keV above the underlying bremsstrahlung x-ray continuum.

**Fig. 14:**
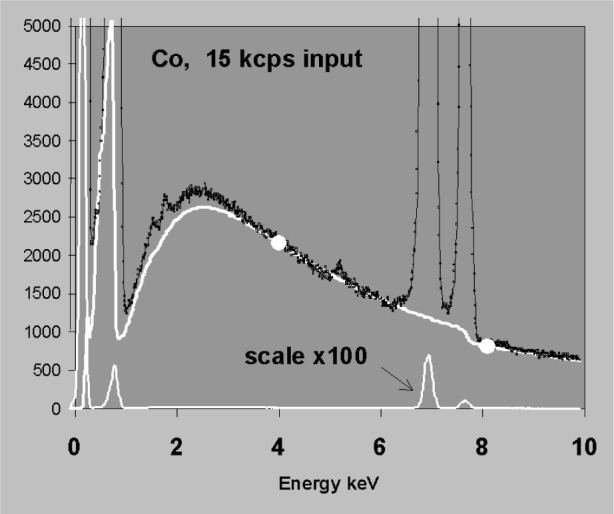
Spectrum from pure Cobalt standard obtained at 20 kV at modest count rate. The theoretical background fitted to points near 4 keV and 8.2 keV (white curve) gives a fairly good estimate throughout the energy range.

**Fig. 15:**
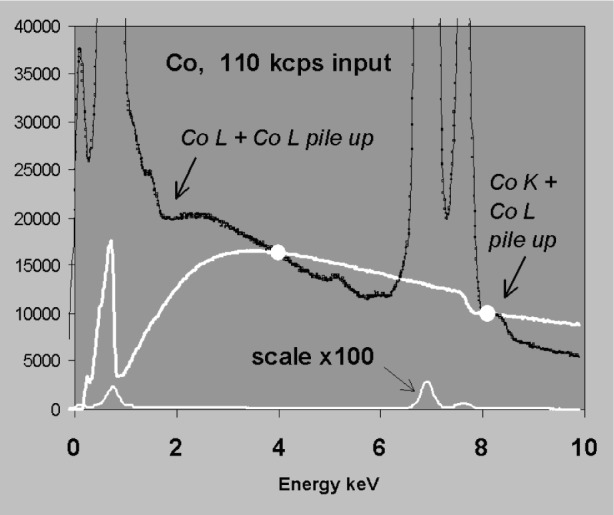
Spectrum from pure Cobalt standard obtained at 20 kV at high input count rate using identical fitting regions to Fig. 14. The pile-up continuum extending below the CoK+CoL sum peak produces a bad estimate of background.

Another potential source of a smooth background artifact is pile up which occurs at high count rates. Most EDXS systems are fitted with pile up inspectors that are fast and effective for high energy photons. However, lower energy photons are more difficult to detect in the presence of noise. Therefore, at high rates a pile up continuum can appear in the spectrum as a tail on the high energy side of peaks and a sum peak may appear at the end of the tail [13]. Figure 14 shows a good background fit to a spectrum from pure cobalt at a modest count rate. In particular, note the presence of a strong CoL peak well below 1 keV. When the pulse processor setting is switched to a shorter time constant and the beam current increased, the high count rate spectrum shown in Fig. 15 exhibits strong pile up effects involving CoL photons. As a consequence, the same background modelling procedure now gives a very poor estimate of background.

Thus, background modelling can in principle produce the most accurate background correction but it is particularly sensitive to the choice of points for fitting. The digital filtering approach is less sensitive to smooth background artifacts and avoids the need to choose fitting points so has more widespread applicability and gives more reproducible results. Both approaches require considerable care in implementation.

### 2.5 Detection Limits

If a peak is isolated and on a fairly flat background as in Fig. 6 then the background can be fitted either side of the peak, interpolated, and subtracted to give a very accurate measure of total peak area. This is the ideal situation referred to in most textbook calculations of statistical detection limits. However, Figs. 10 to 15 demonstrate how systematic errors in background subtraction can exceed the statistical fluctuations from channel to channel. In addition, if peaks overlap, the background for a given peak is effectively raised by its neighbour. Not only does this worsen the statistical detection limit [14] but also any systematic error in overlap correction may become the limiting factor in reliable detection of low concentrations.

## 3. Peak Overlap Correction

### 3.1 Overlap Factors

Isolated peaks are rarely found in routine analysis. For example, Kβ/Kα overlap of the transition elements is commonplace in analysis of steels and combinations such as WM/TaM/SiK, and PbM/SK involve closely overlapping lines. If overlapping peaks have at least some energy regions where there is no overlap, the interference can be dealt with using “overlap factors” [3]. To do this, a spectrum from a standard containing just one elemental peak is acquired, then the net window integral for this peak and all the other elemental peaks is obtained from this spectrum. Thus, the relative fraction of the window integral picked up in the energy windows for other elements can be determined. A complete matrix of factors can be constructed provided enough pure elements or simple compounds are available to obtain the factors for each element. When the unknown spectrum is recorded, the net integral in each energy window is equated to the sum of the window integral for the element of interest plus some fraction of all the other elemental peaks present. The set of simultaneous equations is solved to find the net integrals in the absence of overlaps.

The overlap factor approach avoids any detailed knowledge of peak shape but requires a considerable amount of experimental work to characterise a particular system. If the x-ray detector is changed, or the pulse processing time constant is changed, or the resolution, linearity, zero position, or calibration changes with count rate or degrades over time, then overlap factors will change and corrections will be biased. This is less of a problem in mineralogical analysis where count rates on different materials are similar and there are relatively few elements to consider for analysis. The approach is thus suited to dedicated analysis tasks involving a fixed set of known elements but generally gives a very poor result with severe overlaps such as PbM/SK, BrL/AlK or TaM/WM/SiK. For routine analysis of a wide range of materials, a much more flexible approach is required.

### 3.2 Least Squares Fitting of Experimental Profiles

When the profile for each elemental peak is known, least squares fitting can be used to find the best combination of profiles that match the sample spectrum [8]. Profiles can be experimentally determined using pure element or simple compound standards and the background is subtracted, either explicitly or by digital filtering with a zero-area correlator like the “Top-Hat” [9,10]. The sample spectrum also must have background removed by the same technique and the linear least squares algorithm will then find that combination of profile intensities that gives the minimum sum of squares of differences with the background-subtracted sample spectrum. A refinement to the technique is to give more weight to those channels where the statistical standard deviation is small and the overall statistical precision of the determined intensities can be calculated, even in the more complex case where digital filtering has been used [9,10].

Acquiring a comprehensive library of profiles for all elements involves a lot of time and expense. If x-ray detectors from the same manufacturing process have similar characteristics, then the same profile library can in principle be used with more than one detector. Thus, a set of “virtual standard profiles” can be provided as part of a packaged software solution provided there is some method for correcting for changing resolution and calibration from system to system. In practice, this can be achieved if the system noise is automatically monitored by the electronics; variations in electronic noise can then be corrected by convolving the spectrum and profiles with the appropriate Gaussian functions to bring them all to the same effective noise value. Similarly, zero and gain of the energy scale can be periodically checked using a suitable calibration standard so that the energy scales can be brought into register.

### 3.3 Calculated Peak Profiles and Incomplete Charge Collection

**Fig. 16:**
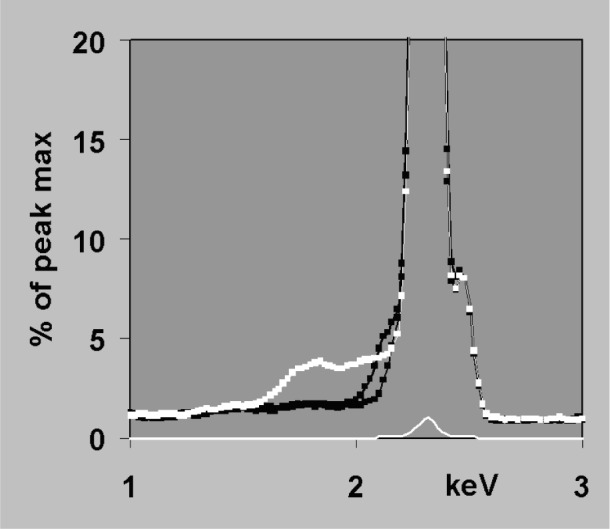
Spectra of FeS_2_ recorded at 20 kV with different detectors and scaled to the sulphur peak. The bremsstrahlung background level is just less than 2 % of the S Kα peak height and the ICC tail contribution can be seen above the background.

**Fig. 17:**
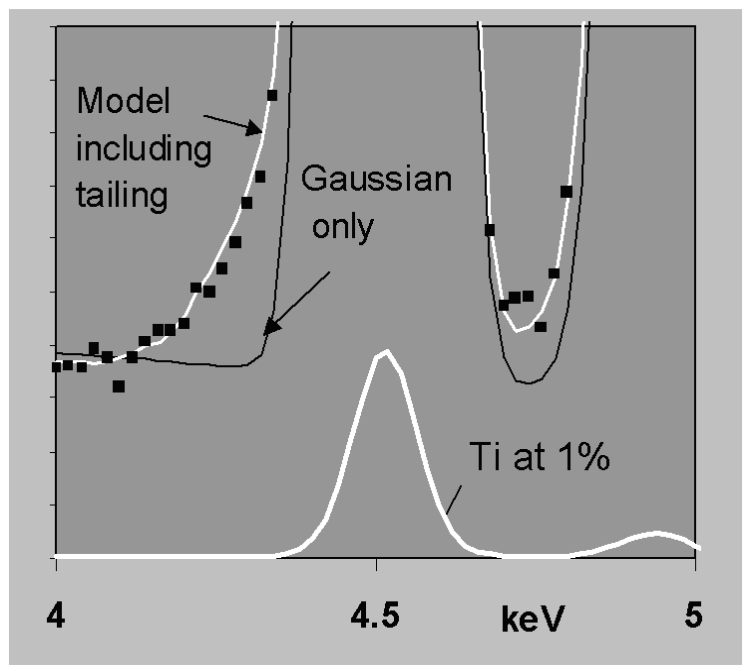
Spectrum from pure Ti at 20 kV shown with two scales differing by a factor of 100. The best Gaussian approximation is shown but the model that includes tailing provides a much better fit to the experimental peak.

Stored experimental profiles effectively characterise the energy response of a particular detector but suitable standards are not always available for every element and profiles also have to be obtained for all the EDXS configurations that are to be used. In principle, a mathematical model can overcome these difficulties. To first approximation, the response function of an x-ray detector to monochromatic radiation of energy *E* is a Gaussian function,

$$G_{i}=\exp(-2.773\cdot {\left[(x_{i}-x_{E})/fwhm_{E}\right]}^{2})\cdot 0.9394\cdot \Delta /fwhm_{E}.$$ (7)

where *x*_i_ is the energy corresponding to channel i and *Δ* is the channel width for the digitised spectrum. If the energy scale is assumed to be linear then

$$x_{E}=x_{0}+g\cdot E$$ (8)

where *x*_0_ is the electronic zero and *g* is the gain. The peak resolution, *fwhm_E_* is given by

$$fwhmE^{2}=fwh{m_{0}}^{2}+dispersio{n_{E}}^{2}$$ (9)

where *fwhm*_0_ refers to the electronic noise contribution and *dispersion_E_* is the detector contribution to spectral resolution. For an ideal detector, *dispersion_E_*^2^ = *k · E*, where *k* is a constant (typically around 2.48 for Si(Li) if energies are all in eV). These approximations work quite well for x-ray detectors in the energy region 4 keV to 15 keV but incomplete charge collection (ICC) near the front contact of the transducer means that in silicon-based detectors, the response function can change significantly for x rays that are absorbed strongly in silicon. In practice this means for energies around 1.84 keV to 3 keV and for energies below 1 keV, ICC effects give peaks a low energy tail, shift the centroid below *x_E_* and make the peak broader than *fwhm_E_* [15,16]. Various theoretical models have been proposed and suitable formulae suggested for representing ICC distortion, [e.g., 17,18]. However, there is no one model that works for all detector designs and the nature and degree of ICC may vary from detector to detector because of changes in the manufacturing process [19] and may also be affected if there is any ice build up on the detector surface. To date, the IEEE “Peak to background” test, which ratios the height of the MnKα peak from an Fe55 radioactive source to the background level at 1 keV, is the only official standard pertaining to charge collection [20] but is a very poor measure of tailing. For example, Fig. 16 shows three spectra of FeS_2_ taken at 20 kV with different x-ray detectors. In the detector showing the worst tailing on SKα, IEEE *P*:*B* was 18 000:1 whereas the one with the least tailing gave P:B = 16,000:1. Either value would normally be regarded as an indicator of excellent charge collection and in this case, the better detector had a worse value of *P*:*B*. At energies > 3 keV, the degree of tailing is less because x rays penetrate deeper into Si and some of the effect of ICC can be taken into account by shifting and broadening a standard Gaussian function. However, some tailing remains and Fig. 17 shows that even with a good detector with ICC performance equivalent to the best in Fig. 16, the residual non-Gaussian tail component still represent of the order of 1 % of the main Ti Kα peak. Tailing can be accommodated by a suitable modification to the shape model as shown in Fig. 17. Any uncorrected residual tail may appear in the results as spurious concentrations for elements not present in the sample. Furthermore, as shown in Fig. 11, a residual tail can affect accuracy of background subtraction if background modelling is used.

### 3.4 The Effect of Inaccuracies in Position and Width

Even if the peak profiles include ICC, either because they have been recorded experimentally or have been accurately modelled, then the position and width may still vary due to instrumental effects. Peak position is normally easier to control and a good design with precision components can deliver stabilities < 0.01 %/°C for gain, *g*. However, stabilisation for zero, *x*_0_, is not available on all electronic pulse processors and the baseline may move with count rate, particularly if there are a lot of low energy x rays that go undetected by the electronic pile-up inspector [21]. The electronic noise, *fwhm*_0_ in Eq. (9), will change with processing time constants used in the electronics and may also change with count rate. In fact some electronic processors have adaptive shaping that is designed to produce the best resolution possible for a given count rate [22]; this not only guarantees that resolution changes with count rate, but also introduces a weighted series of Gaussians with different *fwhm*_0_ values into the peak shape model. Noise contribution to a detector may also change over time and be temporarily affected by electronic interference. It is therefore imperative to have a some method of correcting for inevitable changes in position and resolution.

**Fig. 18:**
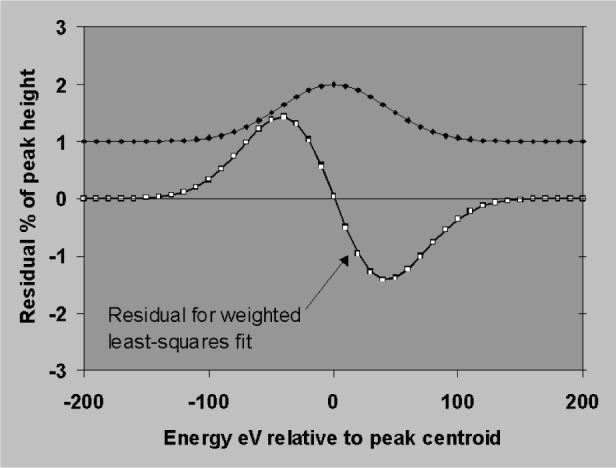
Residual after fitting a profile shifted by 1 eV relative to a peak, *fwhm* = 100 eV, on a background of the same height.

**Fig. 19:**
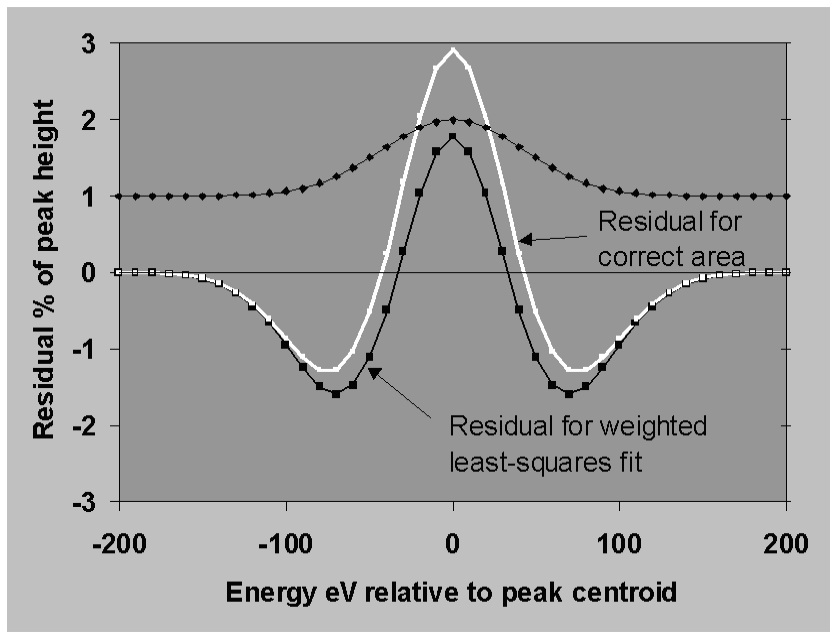
Residual after fitting a profile 3 eV too wide to a peak, *fwhm* = 100 eV, on a background of the same height. White curve shows the zero area residual that would result if the fitted area were exactly correct. The area of the least squares residual is negative so the true peak area has been overestimated.

**Fig. 20:**
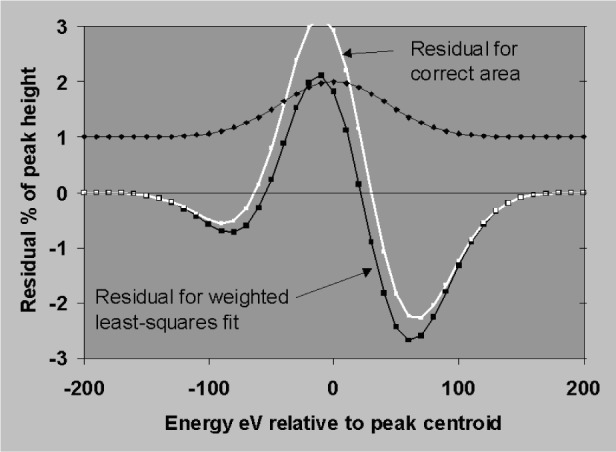
Residual after fitting a profile shifted by 1 eV and 3 eV broader than a sample peak, *fwhm* = 100 eV, on a background of the same height. The least squares residual has negative total area.

The sensitivity to errors in position and width will now be established using the simplifying assumptions that peaks are simple Gaussians, that the background subtraction is perfect and that profiles are fitted to the data by weighted least squares. Figure 18 shows the result of fitting a profile with correct *fwhm* = 100 eV but shifted by 1 eV relative to a sample peak on a background of the same height (*P*/*B* = 1). The residual is obtained by subtracting the background and the fitted profile result. The residual shows a characteristic bipolar shape with lobe amplitudes of about 1 % of the original peak height for a positional error of 1 % of *fwhm*. The residual has close to zero net area so that the area of the fitted peak is still correct provided the shift is a small percentage of *fwhm*. Figure 19 shows the result of fitting a profile with the wrong width, *fwhm* = 103 eV, to a peak with *fwhm* = 100 eV on a background of the same height. The least squares algorithm guarantees that the residual has the minimum weighted sum of squares but when the resolution is incorrect, the residual has net negative area. This means that even with an isolated peak and an easy background subtraction, the area (and therefore measured x-ray line intensity) will be overestimated when the profile used for fitting is too broad. In practice, there are likely to be both position and width errors and Fig. 20 shows an example where not only is the area of the peak overestimated, but also the residuals might be mistaken as small concentrations of elements with nearby peaks.

Statistical weighting does have an influence on the sensitivity to error. If a peak is very small compared to the background, then statistical weighting is uniform and the results are the same as if no weighting was used. If the peak-to-background ratio is very high, then statistical weighting will force the residual to be small in the wings of a peak and this produces a different result. Other techniques for peak area measurement are affected to some extent by shift and width errors. For example, if a peak is integrated over an energy window close to *fwhm* in width, the area represents a certain proportion of the true peak area. If the spectrometer drifts or resolution changes, then this proportion will change.

**Fig. 21:**
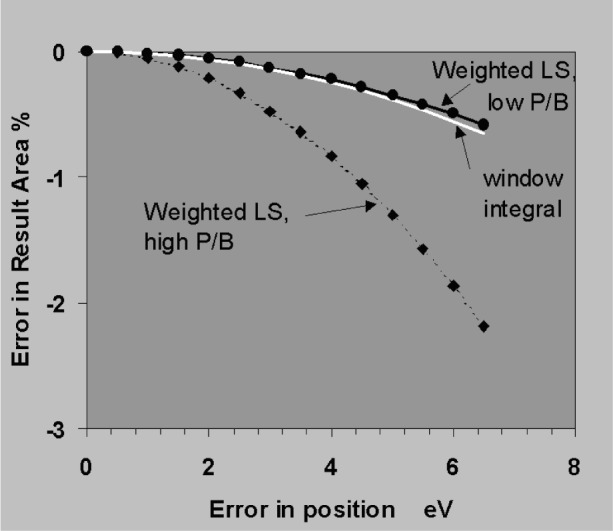
Relative error in measured peak area for an isolated peak of *fwhm* = 100 eV when there is a positional error. For the general case, the horizontal axis can also be regarded as the shift as a percentage of *fwhm*.

**Fig. 22:**
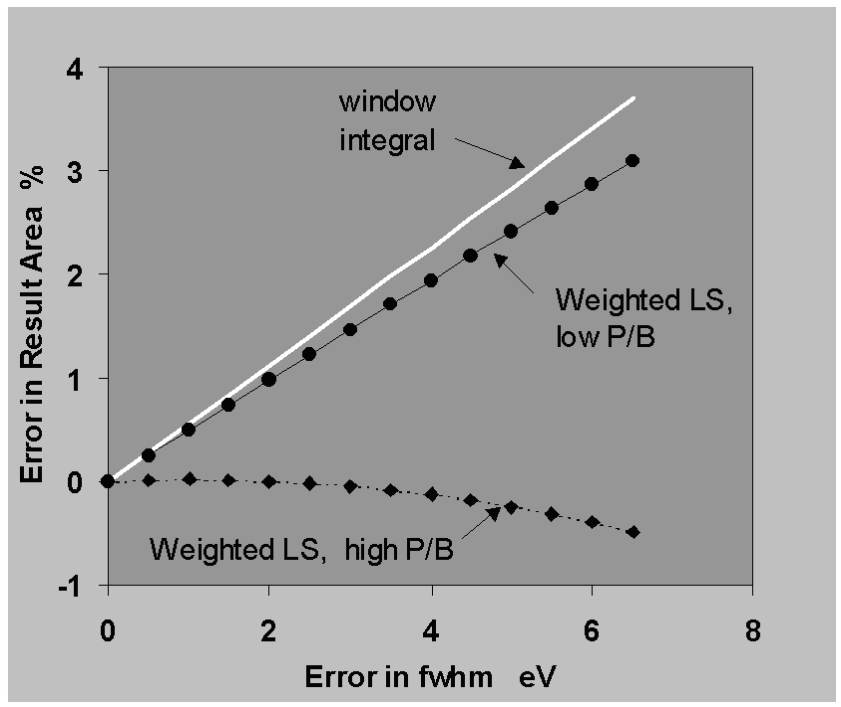
Relative error in measured peak area for an isolated peak of *fwhm* = 100 eV when there is error in the *fwhm* used for the fitted profile or *fwhm* used for window integral. For the general case, the horizontal axis can also be regarded as a percentage of *fwhm*.

In Fig. 21 the sensitivity to shift errors is compared for simple window integral and least squares fitting techniques. For an isolated peak, a small shift in position has hardly any effect on the measured area and even in the extreme weighted least squares case, a shift of 4 % of *fwhm* gives less than 1 % relative error in the measured intensity. In contrast, an error in peak width has a significant effect on peak areas as shown in Fig. 22. The case of weighted least squares with an isolated high peak on a low background will rarely occur so it is the curves for the window integral method and for peaks on a high background that are more likely to be relevant to practical situations of analytical interest. These show that for a 4 % error in *fwhm*, the relative error in area is about 2 % and a useful approximation is that the relative error in measured area is about half the relative error in fwhm assumed for the peak.

**Fig. 23:**
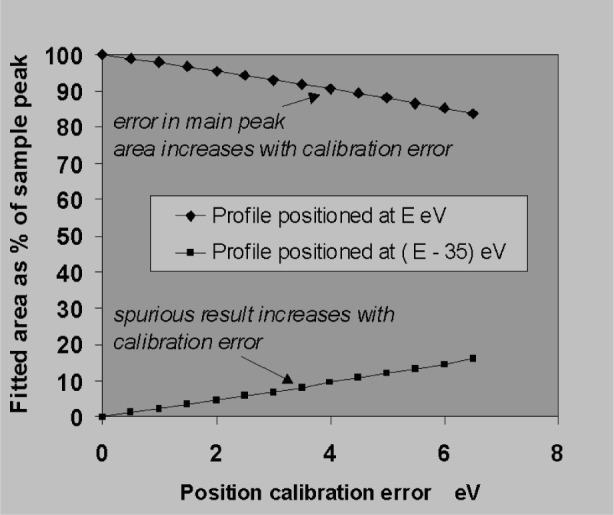
Effect of position error when two peak profiles are fitted to a single peak, *fwhm* = 100 eV at energy *E* eV.

A much more common and serious problem for EDXS is the case of overlapping peaks. When peaks are less than a *fwhm* apart, a small error in position will seriously affect the distribution of areas in the fitted result. For example, if there was a large peak near 1.75 keV in the spectrum and it was not known whether there was some Si or W present, two profiles would be fitted to the spectrum corresponding to the expected positions of SiKα and WMα which are 35 eV apart. Figure 23 demonstrates the effect of a positional calibration error on the fitted results when the true peak contains only WMα. When there is no positional error then the result is the expected unity mass fraction for W, 0 for Si. However, if the profiles are both too high in energy, the W is underestimated and a spurious concentration of Si is reported. In these extreme overlap situations, a useful rule of thumb is that the spurious contribution as a fraction of the main peak is given by the ratio of Shift/Separation. Thus, for two peaks separated by 40 eV, a shift error of 4 eV gives an error in area for both peaks equivalent to about 10 % of the main peak area. Therefore, in an even more extreme overlap example such as AlKα/BrLα, where the separation is only 7 eV, just a 1 eV error in position will produce errors equivalent to 14 % of the main peak area.

Shift and resolution errors can still be a problem in more modest overlap situations. As Fig. 20 suggests, if there are any peaks within ± 2 × *fwhm* of a large peak, then the residual due to position or width errors can be picked up as spurious concentration. As shown in Sec. 2.4, background modelling is subject to errors particularly when suitable background points cannot be found near to the peak being measured. The digital filtering approach avoids this problem because the “Top-Hat” effectively makes a local background estimate very close to the peak. However, if there are errors in position or width, the sort of residual shown in Fig. 20 now influences the “Top-Hat” background estimate for nearby peaks so the influence of these errors extends to ± 3 × *fwhm* from the main peak. For example, MgKα and AlKα peaks are separated by 230 eV and if a trace of Mg is to be determined in the presence of a large Al concentration, then position and width errors can still be an issue if the *fwhm* exceeds 80 eV.

### 3.5 Nonlinear Fitting and Derivative Profiles

**Fig. 24:**
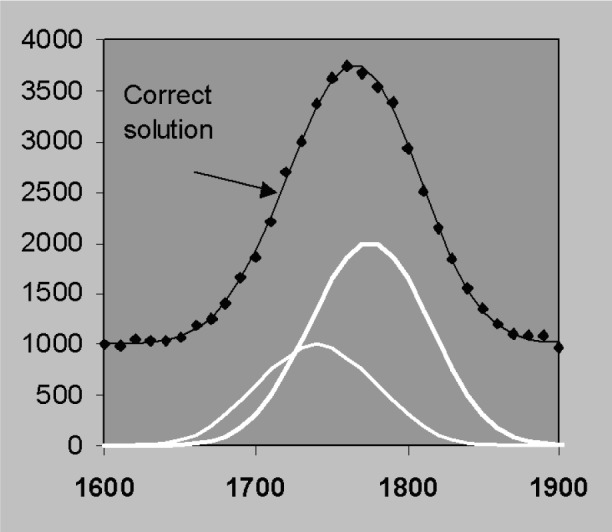
Example channel count data where the two peaks shown in white, *fwhm* = 100 eV and separated by 30 eV are superimposed on a uniform background and the data points show the effect of Poisson counting statistics. The left peak is half the height of the right peak and the correct solution is shown as a smooth curve through the data points.

**Fig. 25:**
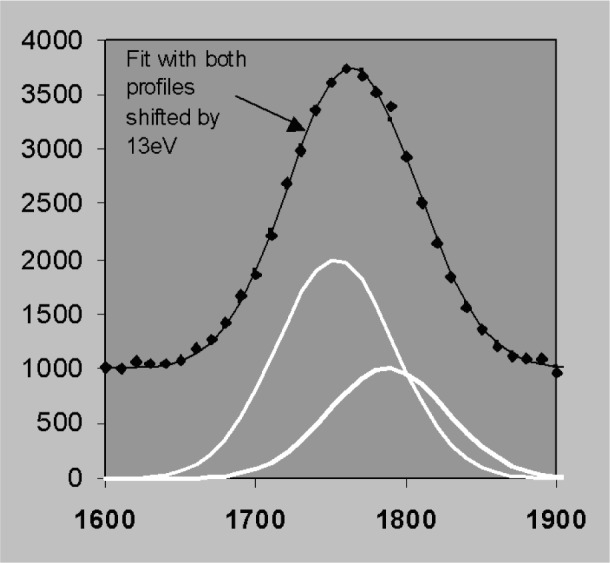
Same data as for Fig. 24 overlaid with the fitted result when the two candidate profiles are shifted by 13 eV. Although the solution is totally incorrect, the fitted result is still a good fit to the data points.

**Fig. 26:**
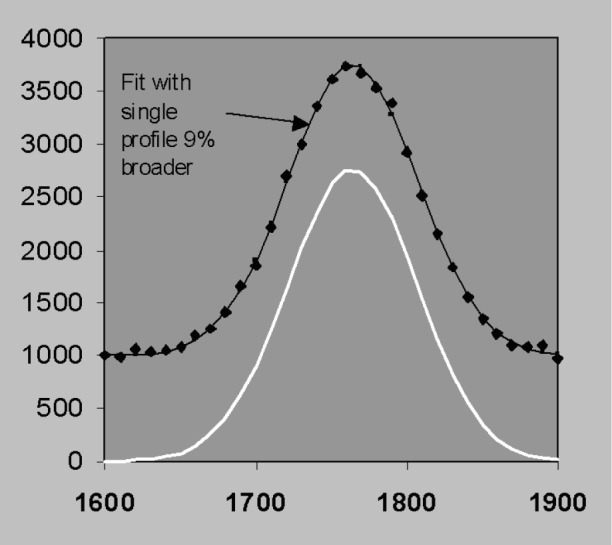
Same data as for Fig. 24 overlaid with the fitted result when a single broader profile shifted by 24 eV is used. Again the fitted result is a good fit to the data points even though the solution is totally incorrect.

The discussion so far has assumed that peak width and position are predetermined in which case the least squares solution can be obtained in closed form without iteration. Position and width can also be included as parameters in a either a non-linear least squares fit by sequential simplex for example [23] or by including first and second derivative profiles in a linear fit [24]. However, if we let position and resolution vary without constraint, the best fit to the data can be obtained with combinations of position and resolution that do not correspond to the real solution [25]. This problem occurs because statistical noise can make it impossible to differentiate between plausible alternatives as the following example will demonstrate. Figure 24 shows a mixture of two Gaussian peaks, *fwhm* 100 eV, separated by 30 eV on a uniform background. This could correspond to a TaM peak next to a SiK peak for example and a representative level of statistical noise is shown. If the candidate peak profiles are allowed to shift along the energy axis while maintaining a constant separation, then another good fit is obtained with a shift of 13 eV as shown in Fig. 25. In this case, even though the fit is good, the peak heights are completely reversed and the fitted result for the left peak is now twice the height of the peak on the right! If the profile width is also allowed to vary, then another good fit can be obtained by fitting a single peak to the sum as shown in Fig. 26. In this case, if the sample did contain Ta and Si, the good fit with just a single peak would suggest that only one of these elements were present. Therefore, even qualitative identification of elements present would fail.

Whereas varying position and width can be used to obtain a good fit to a single peak, it is not suitable for resolving severe overlaps. If there are always going to be strong isolated peaks present in the spectrum and only *x*_0_, *fwhm*_0_ and *g* are varied in the fitting procedure, then the dominant peaks will effectively provide an internal calibration and constrain the fit to provide sensible solutions [26]. However, in low kV spectra in particular, there are unlikely to be any strong isolated peaks. Furthermore, if the spectrum contains only a few counts, non-linear methods will not converge on any sensible result because residual errors in the fit will be totally masked by statistical noise. Therefore, for an entirely general solution to microanalysis, a reliable energy calibration and specific measurement of resolution are required in order to overcome the accuracy barriers described in Sec. 3.4.

### 3.6 Calibration Requirements

If a Gaussian peak is on a low background, the background can be subtracted by linear interpolation and a quadratic curve fitted to the log intensity by weighted least squares using all points above 10 % of peak maximum. Thus, Eq. (8) can provide estimates for *x_E_* and *fwhm_E_*. If the area of the peak is *N* counts, then the standard deviation in measured position is approximately *σ_x_* = 0.43 *fwhm_E_*/*sqrt*(*N*) and the standard deviation in measured resolution is approximately *σ_fwhm_* = *fwhm_E_*/*sqrt*(*N*). (These values have been confirmed by simulation and are close to the precision obtainable using the full peak [27]). With two Gaussian peaks, A and B corresponding to energies *E*_A_ and *E*_B_, the fitted values of position, *x*_A_ and *x*_B_ can be used to determine *x*_0_ and *g* using Eq. (8). Then the predicted peak position for a line at energy *E* will be

$$X_{E}=x_{A}\cdot (1-p)+x_{B}\cdot p$$ (10)

where *p* = (*E − E*_A_)/(*E*_B_ *− E*_A_). The standard deviation in this predicted position will then be

$$\sigma_{E}={\left[{\sigma_{A}}^{2}\cdot {\left(1-p\right)}^{2}+{\sigma_{B}}^{2}\cdot p^{2}\right]}^{0.5}$$ (11)

For EDXS systems without an automatic zero measurement, calibration requires a spectrum with two well defined x-ray peaks of similar area, usually AlKα and CuKα at 1.49 keV and 8.04 keV. If a spectrum with 300 000 total counts is recorded at 20 kV from a typical Si(Li) detector with *fwhm* = 133 eV at 5.9 keV and there are 50 000 counts in each peak, then the precision in calibration at energy *E* will from Eq. (11) be ± 0.19 eV at the OKα energy. Some EDXS systems have an automatic “strobed” zero energy measurement which effectively provides a reference peak corresponding to zero energy. Therefore, calibration can be achieved using a spectrum with just a single x-ray peak. If this is CuKα and has 100 000 counts (again assuming a total spectrum area of about 300 000 counts) and the strobe zero peak has an area of 100 000 counts, then the precision in calibration at energy *E* will be ± 0.07 eV at the OKα energy. Resolution calibration is achieved in a similar manner where *fwhm*_A_ and *fwhm*_B_ are used to determine *fwhm*_0_ and *k* using Eq. (9). Using standard methods for error propagation, standard deviation for the predicted *fwhm* at energy *E* will be

$$\sigma_{fwhm_{E}}={\left[{\sigma_{fwhm_{A}}}^{2}\cdot {\left(fwhm_{A}/fwhm_{E}\right)}^{2}\cdot {\left(1-p\right)}^{2}+{\sigma_{fwhm_{B}}}^{2}\cdot {\left(fwhm_{A}/fwhm_{E}\right)}^{2}\cdot p^{2}\right]}^{0.5}$$ (12)

Using the same example with a total spectrum area of 300 000 counts the relative uncertainty in *fwhm* for OKα would be 0.89 % for the method using AlKα and CuKα peaks with 50 000 counts and 0.24 % using strobe zero peak and CuKα peak of 100 000 counts.

Given the magnitude of effects demonstrated in Sec. 3.4, it would appear that calibration for position and resolution should not be a barrier to accuracy provided a suitable standard is used and at least 300 000 counts are acquired in the calibration spectrum. If the temperature changes by only 1 °C after the calibration, positional errors could typically be 1 eV or more. Although some sophisticated electronic designs claim high stability, in many systems, variation in position and resolution can be as much as 5 eV between low and high count rate and some electronic designs may also be sensitive to the balance of low and high energy x rays in the spectrum. Consequently, position and resolution errors are more likely to be dominated by instrumental effects rather than the counting statistics for calibration described by Eqs. (11) and (12).

## 4. Total System Stability and Reproducibility

Figures 14 and 15 indicate how the measured spectrum may change when count rate or electronic processor settings are altered. As well as background artifacts, peaks may shift in position and resolution may deteriorate at high count rates so that any calibration performed at low count rate or a particular processor setting may become invalid at higher count rates or a different setting. Figure 16 points out some of the variability in ICC that may be seen from detector to detector so that even at low count rates, the same software and procedure may produce different results with different detectors. Complete EDXS analysis systems are designed for ease of use and it is often difficult to get access to individual components to make specific tests. Furthermore, manufacturers may include specialised correction software to correct for certain types of instability and variation. Often the only way to test for stability and reproducibility is to treat the total system as a “black box” and validate it in real situations that are representative of the analytical problems to be tackled. There will always be some statistical fluctuation in results due to the operation of subtracting background so that both positive and negative results are to be expected for any element with zero concentration. Any system that does not report small negative concentrations should therefore be treated with suspicion. Usually, tests similar to the following can be performed on any EDXS system and can be adapted to suit the particular application.

A severe test of spectrum processing is to acquire a spectrum from a sample of Al or Al_2_O_3_ and force the system to analyse for MgK, AlK, SiK, and BrL. Of course, only Al should be detected, but any error in background modelling or overlap correction will show up as spurious concentrations of Mg, Si, and Br. Since BrL is only 7 eV apart from AlK, this test is extremely sensitive to peak position accuracy whereas Mg and Al provide a check for less severe overlaps. Table 1 demonstrates the test in use. Analysis of an Al_2_O_3_ sample has been performed at different pulse processor settings and at a variety of input count rates. The statistical errors reported by the software package are shown next to each analysis result. Both positive and negative results are reported and any result within ± 3 standard deviations could reasonably be regarded as the result of statistical variation. However, the 35 kcps result at PT3 shows some significant spurious concentrations of Mg, Si, and Br.

The Al/Br overlap is particularly severe but is typical of the L/K overlaps that occur at low energies such as Ti/O, V/O, Cr/O. A less severe test is to use a sample of pure Si and analyse for WM, SiK, TaM, checking for spurious amounts of W or Ta. In this “null” testing approach, it is essential that the system reports the results for all elements and their standard deviations. Then it is possible to establish what the real limit of detectability is for a low concentration of any element. As Fig. 2 shows, analysis at low kV presents a severe challenge for any EDXS system but again, null tests can easily be devised to establish how effective spectrum processing is under these conditions. Even if peak intensities can be determined with some confidence, there is no guarantee that chemical effects will not affect XR-CFs and some testing with representative compounds is always desirable. While it is important to explore and gain confidence in all likely analytical configurations, there is little point in pushing the EDXS to extremes that are never likely to be used.

## 5. Conclusions

Some time in the future, spectrometers may have sufficiently good resolution to leave some background between every characteristic line so that simple interpolation could be used to obtain peak areas and this would be insensitive to peak shape and position errors. Meanwhile, all current EDXS systems have to cope with peak overlap and whatever the sophistication in spectrum processing algorithms, the following factors always affect accuracy in determining characteristic peak intensities:

1. Prediction of peak shape, width and position for every characteristic line
2. Measurement of background intensity
3. Stability of spectrometer characteristics with time and changing count rate

Accuracy with relative errors less than 2 % for major constituents (mass fractions greater than 0.1) and errors less than 0.001 mass fraction at low concentrations can usually be achieved by restricting count rates to below 3 kcps, selecting specific elements on the basis of prior knowledge of the sample, working at 15 kV or higher so that the well-separated K lines for transition elements are available, avoiding analysis of any line below 1 keV in energy and making regular checks of energy calibration and beam current. To achieve this accuracy beyond this restricted range of application, particularly towards low keV, requires excellent electronic and detector stability and improved methods for modelling peak and background shape.

## Figures and Tables

**Table 1:** Black box testing using spectra from Al_2_O_3_ sample obtained in SEM at 20 kV and various beam currents. Results for mass fraction with statistical standard deviation estimates are shown for various electronic settings for process time (PT) and input count rates. Statistically significant “mistakes” are shown in italics

Spectrum	Mg	Al Mass fraction	Si	Br
PT6, 1 kHz	−0.07±0.13	53.90±2.13	0.08±0.21	−1.90±3.35
PT5, 10 kHz	0.04±0.02	53.20±0.41	0.07±0.04	−0.73±0.65
PT3, 1 kHz	0.13±0.15	48.71±2.87	0.11±0.30	7.51±4.44
PT3, 10 kHz	0.16±0.03	53.84±0.56	0.08±0.05	−2.15±0.88
PT3, 35 kHz	*0.19±0.02*	54.48±0.31	*0.15±0.03*	*−3.58±0.49*

## References

[1] Castaing R (1951). Application des sondes electroniques a une methode d’analyse ponctuelle chimique et cristallographique. Thesis.

[2] Reed SJB (1993). Electron Microprobe Analysis.

[3] Reed SJB, Ware NG (1973). Quantitative Electron Microprobe Analysis Using a Lithium Drifted Silicon Detector. X-Ray Spectrometry.

[4] Dunham AC, Wilkinson FCF (1978). Accuracy, Precision and Detection Limits of Energy-dispersive Electron-microprobe Analyses of Silicates. X-Ray Spectrom.

[5] Beaman DR, Solosky LF (1972). Accuracy of Quantitative Electron Probe Microanalysis with Energy Dispersive Spectrometers. Anal Chem.

[6] Newbury DE (1999). Standardless Quantitative Electron-Excited X-ray Microanalysis by Energy-Dispersive Spectrometry: What Is Its Proper Role?. Microsc Microanal.

[7] Boyes ED, Hartmann IR, Gooding F, Sokola D, Hanna L, Smith DL (1991). EDX chemical microanalysis of bulk specimens in the SEM at low beam voltage and high spatial resolution. Inst Phys Conf Ser No 119, EMAG 91 Bristol.

[8] Statham PJ (1976). A Comparative Study of Techniques for Quantitative Analysis of the X-Ray Spectra Obtained with a Si(Li) Detector. X-Ray Spectrom.

[9] Schamber FH A modification of the linear least-squares fitting method which provides continuum suppression.

[10] Statham PJ (1977). Deconvolution and Background Subtraction by Least-Squares Fitting with Prefiltering of Spectra. Anal Chem.

[11] Lifshin E (1974). The use of solid state x-ray detectors for obtaining fundamental x-ray data. Proc 9th Ann Conf Microbeam Analysis Society.

[12] Fiori CE, Myklebust RL, Heinrich KFJ, Yakowitz H (1976). Prediction of Continuum Intensity in Energy-Dispersive X-Ray Microanalysis. Anal Chem.

[13] Statham PJ (1977). Pile-Up Rejection: Limitations and Corrections for Residual Errors in Energy-dispersive Spectrometers. X-Ray Spectrom.

[14] Statham PJ, Williams DB, Goldstein JI, Newbury DE (1995). Quantifying benefits of resolution and count rate in EDX microanalysis. X-Ray Spectrometry in Electron Beam Instruments.

[15] Statham PJ (1981). X-Ray Microanalysis with Si(Li) detectors. J Microsc.

[16] Craven AJ, McHardy CP, Nicholson WAP (1987). The effect of incomplete charge collection on the peak shapes from Si(Li) x-ray detectors. Proc EMAG 87, Manchester, UK 1987, Inst Phys Conf Ser No 90.

[17] Joy DC, Williams DB, Goldstein JI, Newbury DE (1995). Modelling the Energy Dispersive X-ray Detector. X-Ray Spectrometry in Electron Beam Instruments.

[18] Campbell JL, McDonald L, Hopman T, Papp T (2001). Simulations of Si(Li) x-ray detector response. X-Ray Spectrom.

[19] McCarthy JJ, Williams DB, Goldstein JI, Newbury DE (1995). The Effect of Detector Dead Layers on Light Element Detection. X-Ray Spectrometry in Electron Beam Instruments.

[b20-j76sta] 20IEEE Standard Test Procedures for Semiconductor X-Ray Energy Spectrometers, ANSI/IEEE Std 759–1984 (1984)

[21] Newbury DE, Williams DB, Goldstein JI, Newbury DE (1995). Artifacts in Energy Dispersive X-Ray Spectrometry in Electron Beam Instruments Are Things Getting Any Better?. X-Ray Spectrometry in Electron Beam Instruments.

[22] Mott RB, Friel JJ, Williams DB, Goldstein JI, Newbury DE (1995). Improved EDS Performance with Digital Pulse Processing. X-Ray Spectrometry in Electron Beam Instruments.

[23] Fiori CE, Myklebust RL, Gorlen K (1981). Sequential simplex: a procedure for resolving specral interference in energy dispersive x-ray spectrometry. Proc Workshop on Energy Dispersive X-Ray spectrometry.

[24] Kitazawa T, Shuman H, Somlyo AP (1983). Quantitative electron probe analysis: problems and solutions. Ultramicroscopy.

[25] Statham PJ (1978). Pitfalls in Linear and Non-linear Profile-fitting Procedures for Resolving Severely Overlapped Peaks. X-Ray Spectrom.

[26] Nullens H, Van Espen P, Adams F (1979). Linear and non-linear peak fitting in energy-dispersive x-ray fluorescence. X-Ray Spectrom.

[27] Raznikov V, Dodonov AF, Lanin EV (1977). Data acquisition and processing in high resolution mass spectrometry using ion counting. Internatl J Mass Spectrom Ion Phys.

